# Anode Material Options Toward 500 Wh kg^−1^ Lithium–Sulfur Batteries

**DOI:** 10.1002/advs.202103910

**Published:** 2021-11-16

**Authors:** Chen‐Xi Bi, Meng Zhao, Li‐Peng Hou, Zi‐Xian Chen, Xue‐Qiang Zhang, Bo‐Quan Li, Hong Yuan, Jia‐Qi Huang

**Affiliations:** ^1^ School of Materials Science and Engineering Beijing Institute of Technology Beijing 100081 China; ^2^ Advanced Research Institute of Multidisciplinary Science Beijing Institute of Technology Beijing 100081 China; ^3^ Beijing Key Laboratory of Green Chemical Reaction Engineering and Technology Department of Chemical Engineering Tsinghua University Beijing 100084 China

**Keywords:** high energy density, lithium metal anodes, lithium–magnesium alloys, lithium–sulfur batteries, pouch cells

## Abstract

Lithium–sulfur (Li–S) battery is identified as one of the most promising next‐generation energy storage systems due to its ultra‐high theoretical energy density up to 2600 Wh kg^−1^. However, Li metal anode suffers from dramatic volume change during cycling, continuous corrosion by polysulfide electrolyte, and dendrite formation, rendering limited cycling lifespan. Considering Li metal anode as a double‐edged sword that contributes to ultrahigh energy density as well as limited cycling lifespan, it is necessary to evaluate Li‐based alloy as anode materials to substitute Li metal for high‐performance Li–S batteries. In this contribution, the authors systematically evaluate the potential and feasibility of using Li metal or Li‐based alloys to construct Li–S batteries with an actual energy density of 500 Wh kg^−1^. A quantitative analysis method is proposed by evaluating the required amount of electrolyte for a targeted energy density. Based on a three‐level (ideal material level, practical electrode level, and pouch cell level) analysis, highly lithiated lithium–magnesium (Li–Mg) alloy is capable to achieve 500 Wh kg^−1^ Li–S batteries besides Li metal. Accordingly, research on Li–Mg and other Li‐based alloys are reviewed to inspire a promising pathway to realize high‐energy‐density and long‐cycling Li–S batteries.

## Graphical abstract

Systematic evaluation and summary on using Li metal or Li‒based alloys to construct 500 Wh kg^−1^ Li–S batteries is carried out based on a three‐level quantitative analysis method. A highly lithiated lithium–magnesium (Li–Mg) alloy is capable to achieve the 500 Wh kg^−1^ target besides Li metal.

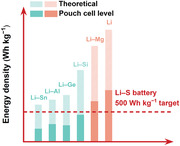

## 1 Introduction

Massive applications of renewable energy to substitute fossil fuels constitute the core issue to tackle climate change and environmental pollution as well as to realize sustainable development and global carbon neutralization. Electrochemical energy storage devices represented by rechargeable batteries have demonstrated great potential for high‐efficient energy storage and conversion for employing renewable energy sources.^[^ ^1^ ^]^ Especially, lithium‐ion batteries (LIBs) have been widely used in electric vehicles, portable electronic devices, and smart grids due to the advantages of high energy efficiency, long cycling performance, reliable safety, and energy density up to 300 Wh kg^−1^.^[^ ^2^ ^]^ However, LIBs are difficult to achieve higher energy density above 400 Wh kg^−1^ due to the capacity limitation of the intercalation electrode materials.^[^ ^3^ ^]^ To realize next‐generation rechargeable batteries with an energy density higher than 500 Wh kg^−1^ according to the Battery500 Consortium plan, new battery systems with high‐specific‐capacity electrode materials are needed to fulfill future applications.^[^ ^4^ ^]^

With a theoretical energy density of 2600 Wh kg^−1^, lithium–sulfur (Li–S) batteries are regarded as one of the most promising next‐generation battery technologies for achieving high actual energy density.^[^ ^5^ ^]^ Specifically, the S cathode possesses merits of high theoretical specific capacity of 1672 mAh g^−1^, low cost (300 $ per ton), and environmental friendliness.^[^ ^6^ ^]^ As for anode material, Li metal anode has not only an ultrahigh specific capacity of 3860 mAh g^−1^, which is ten times higher than that of graphite anode (372 mAh g^−1^), but also a very low equilibrium potential (−3.040 V vs the standard hydrogen electrode) to make it the most promising anode for high‐energy‐density battery systems.^[^ ^7^ ^]^ Since the first attempt in 1960s, a large number of studies have been carried out focusing on improving the cathode specific capacity, prolonging the cycling lifespan, and avoiding the polysulfide shuttle in working Li‒S batteries.^[^ ^8^ ^]^ After decades of development, the S cathode is able to deliver nearly 90% of the theoretical specific capacity and long cycling lifespan over 1500 cycles under ideal working conditions with excessive electrolyte (electrolyte volume to sulfur ratio, E/S ratio more than 8 µL mg_S_^−1^), and anode (negative to positive capacity ratio, N/P ratio higher than 20).^[^ ^9^ ^]^

Nevertheless, considering the target of realizing 500 Wh kg^−1^ Li–S batteries, the electrolyte amount and anode excess have to be controlled to a limited level (typically E/S ratio < 5 µL mg_S_^−1^ and N/P ratio < 3).^[^ ^10^ ^]^ Under such harsh working conditions, practical Li‒S pouch cells fail rapidly with limited cycling lifespan generally shorter than 100 cycles.^[^ ^11^ ^]^ Failure analysis on the practical Li‒S pouch cells suggests that Li metal anode failure constitutes the main reason.^[^ ^12^ ^]^ First, Li metal anode experiences huge volume change during repeated stripping and plating due to the intrinsic hostless nature.^[^ ^13^ ^]^ The severe volume change further causes unstable electrode–electrolyte interface and pulverization of electrode, consequently reducing the anode stability.^[^ ^14^ ^]^ Meanwhile, Li has an ultralow standard redox potential and can react with almost all the components in electrolyte to form complicated solid electrolyte interphase (SEI).^[^ ^15^ ^]^ The SEI is electronically insulated but ionically conductive so that the reactions between Li metal and electrolyte can be partially blocked.^[^ ^16^ ^]^ However, the SEI is generally ununiform to render uneven Li deposition and cracking during cycling so that Li metal is again exposed to electrolyte to result in the consumption of active Li and electrolyte.^[^ ^17^ ^]^ Moreover, massive soluble polysulfides released from the S cathode participate in SEI formation to increase the unevenness of SEI and aggravate the corrosion of Li metal anode.^[^ ^18^ ^]^ Consequently, repeated volume change and the corrosion reactions continuously consume active Li and form massive “dead Li” that is detached from the conductive substrate during cycling, leading to poor recyclability of Li–S battery in practice.^[^ ^19^ ^]^ Even worse, the above circumstances will be deteriorated in high‐energy‐density Li–S pouch cells with limited electrolyte and anode reservoir.^[^ ^12^, ^20^ ^]^

To address the above issues of unstable Li metal anode regarding volume change and high reactivity toward electrolyte and polysulfides, employing Li‐based alloys as alternative anode materials affords a promising scheme.^[^ ^21^ ^]^ Concretely, Li can alloy with silicon, tin, magnesium, aluminum, and many other elements and demonstrates electrochemical activity.^[^ ^22^ ^]^ Alloy anodes with higher electrode potential versus Li deposition (0.1–0.6 V vs Li/Li^+^) can relieve the corrosion reactions with polysulfides and other electrolyte components from the thermodynamic point of view.^[^ ^23^ ^]^ Meanwhile, the alloy element can function as a Li host to accommodate Li deposition and accommodate volume change, further stabilizing the electrode morphology and inhibiting electrode pulverization.^[^ ^24^ ^]^ Following the above design strategies, there have been several reports on using Li alloys to substitute Li metal that demonstrates improved cycling stability in Li‒S batteries.^[^ ^25^ ^]^ However, the employment of Li‐based alloys as anode materials inevitably results in reduced battery energy density considering higher anode potential and reduced specific capacity due to the alloy component, no matter electrochemically active or not. Therefore, aiming at the target of 500 Wh kg^−1^ Li–S batteries, it is necessary to evaluate the potential of Li metal as well as Li‐based alloys to balance the requirement of both energy density and cycling stability. Such theoretical evaluation is expected to afford an important guidance to select suitable anode materials to construct high‐energy‐density Li–S batteries.

In this contribution, we systematically evaluate the potential and feasibility of using Li metal or Li‐based alloys to construct Li–S batteries with an actual energy density of 500 Wh kg^−1^. A three‐level quantitative analysis method is proposed to step by step filter the anode materials by considering the necessary electrolyte amount for the target energy density of 500 Wh kg^−1^. At the first level, only the weight of active materials is considered and the electrode specific capacity is set to be the theoretical value. At the second level, electrolyte is taken into account as the key criterion and the electrode specific capacity is set to be the experimentally reported values. At the third level, the energy density is calculated based on all the components of a Li–S pouch cell including current collectors and packages. Following the above method, ten anode materials are considered and only Li‒Mg alloy demonstrates the feasibility to realize 500 Wh kg^−1^ Li–S pouch cells besides Li metal. Accordingly, recent researches on Li–Mg and other Li‐based alloys are discussed and a perspective is afforded. This work is expected to inspire a promising pathway to realize high‐energy‐density and long‐cycling Li–S batteries and encourage the following attempts on alternative Li‐based alloy anodes beyond Li metal.

## 2 Three‐Level Anode Material Evaluation for 500 Wh kg^−1^ Li‒S Batteries

Considering the intrinsic advantage of Li–S batteries lies in the high theoretical energy density, the key criteria of selecting the anode material for Li–S batteries is the potential and feasibility to realize the targeted energy density of 500 Wh kg^−1^. The energy density of a cell (*E*, Wh kg^−1^) is calculated by the following equation:

(1) $$E=\int \frac{U(Q)}{m_{t}}dQ$$

where *m*_t_ is the total weight of all the components of the cell, *Q* is the discharge capacity, and *U*(*Q*) is the battery voltage, which is the function of *Q*. To simplify the calculation, the average cell voltage $\bar{U}-\bar{U_{\mathrm{de}}}$ is used to substitute *U*(*Q*), where $\bar{U}$ is the average discharge potential of the S cathode and $\bar{U_{\mathrm{de}}}$ is the average delithiation potential of the anode (both vs Li/Li^+^). Considering all the Li source comes from the anode side, the cathode capacity is generally set to be lower than the anode capacity to guarantee full conversion of the sulfur cathode in theory, thus the cell discharge capacity *Q* can be substituted by the sulfur cathode discharge capacity *Q*_S_ = *q*_S_ *m*_S_, where *q*_S_ is the specific capacity of S and *m*_S_ is the mass of S of the cathode. Accordingly, Equation (1) can be rewritten as follows:

(2) $$E=\frac{\left(\bar{U}-\bar{U_{\mathrm{de}}}\right)q_{S}m_{S}}{m_{t}}$$

Equation (2) is used to calculate the energy density that an anode material can potentially reach. Notably, we use the configuration of cathode with elemental S and lithiated anode considering such configuration is widely employed in practice. The anode specific capacity and average delithiation potential are therefore determined at the lithiated state.

Considering the complicated electrochemical behaviors of the electrode materials under working conditions, we first evaluate the anode materials at the ideal material level to determine whether an anode material can potentially realize the targeted energy density of 500 Wh kg^−1^ under very ideal conditions. The cathode and anode potentials are set to be the thermodynamic equilibrium potentials and no kinetic overpotential is considered. The specific capacity of the electrode materials is also set to be the theoretical value regardless of the capacity loss under working conditions. The N/P ratio is set as 1 so that there is no excess anode. Under such ideal conditions, the total mass only involves the cathode and anode active materials and does not consider conductive additives, binders, current collectors, packages, or electrolyte. The total mass *m*_t_ considered in this level is therefore determined as follows:

(3) $$m_{t}=m_{\mathrm{an}}+m_{S}$$

where *m*_an_ and *m*_S_ are the mass of the lithiated alloy anode and the S cathode, respectively, and *m*_an_ *q*_an_ = *m*_S_ *q*_S_ to meet the assumption of N/P ratio = 1.

Ten anode materials are selected to be evaluated at the first level. Besides Li metal, there are seven Li‐based alloys regarding lithium‒magnesium (Li‒Mg), lithium‒aluminum (Li‒Al), lithium‒silicon (Li‒Si), lithium‒germanium (Li‒Ge), lithium‒tin (Li‒Sn), lithium‒bismuth (Li‒Bi), and lithium‒antimony (Li‒Sb) taken into consideration according to the nature of Li metal alloying. Lithiated graphite (LiC_6_) and lithium titanium oxide (Li_7_Ti_5_O_12_) are also included as typical anode materials for LIBs and there are several reports on using them as the anode materials for Li–S batteries.^[^ ^26^ ^]^ **Table** **1** lists the theoretical specific capacity and equilibrium potential of the electrode materials. Among them, Li‒Mg alloy has no limitation of Li content according to the phase diagram.^[^ ^27^ ^]^ Therefore, Li_9_Mg is adopted to represent the binary alloy considering the high Li content and stable alloy structure based on the literature reports.^[^ ^28^ ^]^ Li has a wide solubility in Al to form Li‒Al alloy. However, the maximum charge content of Li is 50 at% limited to the (*α* + *β*) phase according to the Li‒Al binary phase diagram due to the slow diffusion rate of Li at room temperature.^[^ ^29^ ^]^ Therefore, the charged state of Li‒Al alloy is determined to be LiAl.

**Table 1:** Theoretical specific capacity and equilibrium potential of the electrode materials

Charged state	Discharged state	Theoretical specific capacity, *q* [mAh g^−1^]	Equilibrium potential, *U* (vs Li/Li^+^, V)
Li_7_Ti_5_O_12_^[^ ^30^ ^]^	Li_4_Ti_5_O_12_	168	1.6
LiC_6_^[^ ^31^ ^]^	C	340	0.2
Li_3_Bi^[^ ^32^ ^]^	Bi	350	0.8
Li_3_Sb^[^ ^33^ ^]^	Sb	564	0.9
LiAl^[^ ^29a^ ^]^	Al	790	0.4
Li_4.4_Sn^[^ ^22b^ ^]^	Sn	790	0.6
Li_15_Ge_4_^[^ ^34^ ^]^	Ge	1019	0.5
Li_4.4_Si^[^ ^22b^ ^]^	Si	2012	0.4
Li_9_Mg^[^ ^35^ ^]^	Mg	2781	0.1
Li	—	3860	0
S	Li_2_S	1672	2.15

**Figure 1:**
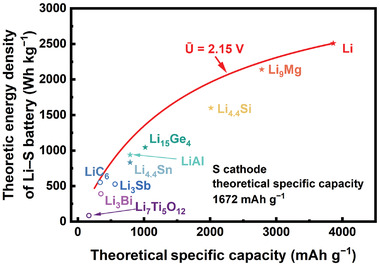
Theoretical energy density of Li‒S battery with different anode materials at the first level of evaluation. The red line is the upper energy density limit at different anode theoretical specific capacities assuming the cell average voltage is 2.15 V.

The estimated energy densities of Li‒S batteries at the first level using the above ten anodes are exhibited in **Figure** **1**. The red line is the upper limit of energy density calculated by adopting the average battery voltage $\bar{U}=2.15\;V$ with Li metal at different theoretical anode specific capacities. According to Figure 1, there are three anode materials regarding Li_3_Sb, Li_3_Bi, and Li_7_Ti_5_O_12_ that cannot afford theoretical energy density higher than 500 Wh kg^−1^. Moreover, considering all the assumptions at the first level, the theoretical energy density with each anode shall be significantly higher than 500 Wh kg^−1^ to ensure the feasibility in practice, thus we choose the theoretical energy density of 800 Wh kg^−1^ as the criteria at this level. Accordingly, six anode candidates regarding Li, Li_9_Mg, Li_4.4_Si, Li_15_Ge_4_, LiAl, and Li_4.4_Sn are taken into next level for evaluation.

At the second evaluation level, namely the practical electrode level, the electrode specific capacity is set to be the reported values based on experiment reports. Necessary components including electrolyte, conductive additive, and binder are taken into consideration to support electron and ion conduction. Other assumptions are similar to the first level including using the equilibrium potentials of the electrode, N/P ratio being 1, and no current collectors or packages. Concretely, the specific capacity, the content of the active material, and the working potential are determined as the highest value according to the references to estimate the upper limit of the energy density that an anode material can reach in a Li–S battery. On the other hand, electrolyte infiltration of the electrodes is necessary especially for the porous electrodes such as the S cathode yet too much electrolyte is a disadvantage for high energy density.^[^ ^36^ ^]^ Therefore, the criterion at this level is whether the electrolyte amount determined by the targeted energy density of 500 Wh kg^−1^ is enough to infiltrate the electrodes in theory.

In the S cathode, elemental S_8_ and the discharge product Li_2_S are electronically insulating (10^−17^ and 10^−30^ S cm^−1^ for S_8_ and Li_2_S, respectively), and they suffer from severe volume change (theoretically 79%) during cycling.^[^ ^37^ ^]^ Therefore, the S cathode is usually constructed by compositing elemental S_8_ with porous conductive carbon with merits of high specific surface area, high electronic conductivity, and light weight to address the above issues.^[^ ^8b^ ^]^ Other sulfur cathode configurations are not considered herein due to their insufficiency in realizing high‐sulfur‐content cathodes and high‐energy‐density Li–S batteries.^[^ ^38^ ^]^ Binders are indispensable for the construction of a practical S cathode based on the above design, and polyvinylidene fluoride (PVDF) is chosen as the binder in cathode due to its wide application.^[^ ^39^ ^]^ Considering the necessity of conductive carbon and binder, the sulfur content of the S cathode is set as 80 wt%, which is considerably high according to the reported values.^[^ ^40^ ^]^ The cathode specific capacity is set to be 1450 mAh g^−1^, which is also a leading value.^[^ ^9c^ ^]^ As for the porosity, Ning et al. found that minimizing the porosity of the S cathode down to 50% exhibited almost no sacrifice of the specific capacity, where the porosity is generally higher than 50% in other reports.^[^ ^36b^ ^]^ Accordingly, the porosity of the S cathode is set as 50%.

**Figure 2:**
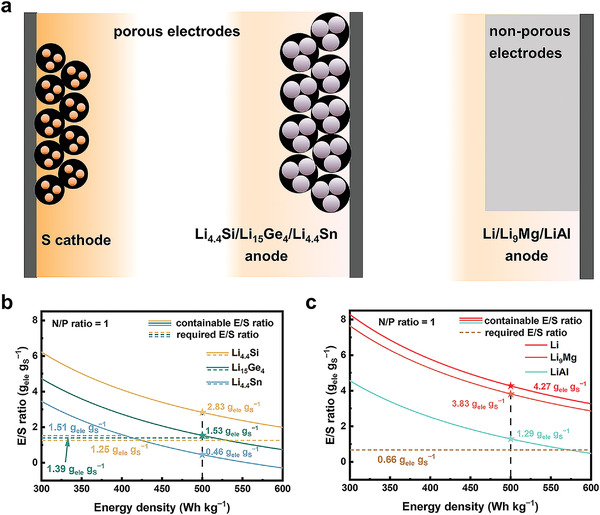
Evaluation on the necessary electrolyte amount to reach the energy density target of 500 Wh kg^−1^ and simultaneously infiltrate the practical electrodes at the second evaluation level. a) Schematic illustration of the structure of practical electrodes. The S cathode and the Li_4.4_Si, Li_15_Ge_4_, and Li_4.4_Sn anodes are porous and require electrolyte infiltration. The Li, Li_9_Mg, and LiAl anodes can be directly used as integral foils. The relationship between the containable E/S ratio and the energy density using b) porous Li_4.4_Si, Li_15_Ge_4_, and Li_4.4_Sn anodes and c) non‐porous Li, Li_9_Mg, and LiAl anodes. The vertical dash lines mark the containable E/S ratio for the target energy density of 500 Wh kg^−1^. The horizontal dash lines mark the required E/S ratio for electrode filtration. If the containable E/S ratio is significantly higher than the required E/S ratio, the corresponding anode material is feasible to reach the energy density target at the second evaluation level.

The Li‐based anodes are classified into two categories according to anode structure. One requires host materials and is therefore porous including Li‒Si, Li‒Ge, and Li‒Sn. The other one is integrated and non‐porous including Li, Li‒Mg, and Li‒Al. Concretely, Li–Si, Li–Ge, and Li–Sn suffer from severe volume change and high internal stress during cycling to render severe electrode pulverization, poor electrical contact, and capacity degradation.^[^ ^7^, ^41^ ^]^ The volume expansion is 320% for Si and 260% for both Ge and Sn after full lithiation to Li_4.4_Si, Li_15_Ge_4_, and Li_4.4_Sn, respectively.^[^ ^22^, ^42^ ^]^ Therefore, Li–Si, Li–Ge, and Li–Sn anodes require porous host materials to relieve volume change similar to the S cathode as is illustrated in **Figure** **2**a.^[^ ^43^ ^]^ The delithiation specific capacities are 1800, 900, and 600 mAh g^−1^ for Li_4.4_Si, Li_15_Ge_4_, and Li_4.4_Sn, respectively.^[^ ^25^, ^44^ ^]^ The contents of the anode active materials are 80 wt% derived from Zhao's report (**Table** **2**).^[^ ^25a^ ^]^ The porosity of the Li‒Si anode is determined according to Heubner's study.^[^ ^45^ ^]^ When the porosity of the Si/C anode at the delithiated state was reduced from 70% to 60% (corresponding to the porosity at the lithiated state reduced from 40% to 10% due to the volume change), the performance degraded about three orders of magnitude.^[^ ^45^ ^]^ Therefore, the porosity for the Li–Si anode is set to be 40% noting the anode is set to be the lithiated state. Unfortunately, there is little research on determining the minimized porosity of Li–Ge and or Li–Sn anodes. Considering their similar properties with the Li–Si anode, the porosity is also set to be 40%.

**Table 2:** Practical electrode performances based on reported values

Electrode	S	Li	Li_9_Mg	LiAl	Li_4.4_Si	Li_15_Ge_4_	Li_4.4_Sn
Practical discharged state	Li_2_S	Li	LiMg_2_	Al	Si	Ge	Sn
Practical specific capacity, *q* [mAh g^−1^]	1450	3860	2626	790	1800	900	650
Active material density, *ρ* [g cm^−3^]	2.1	0.53	0.61	1.73	1.15	2.02	2.55
Active material content, *k* [wt%]	80	100	100	100	80	80	80
Carbon content, *k*_C_ [wt%]	10	0	0	0	10	10	10
Binder content, *k*_B_ [wt%]	10	0	0	0	10	10	10
Porosity *p* [%]	50	0	0	0	40	40	40

As for the second category regarding Li, Li–Mg, and Li–Al, these anodes are free from host materials and can be directly used as integral foils in working batteries. Therefore, the active material content is 100% and the porosity is 0 as shown in Figure 2a.^[^ ^24^, ^46^ ^]^ Unlike Li metal anode that can be fully discharged, full delithiation of Li–Mg alloy from Li_9_Mg to Mg will result in severe performance degradation.^[^ ^47^ ^]^ When the Li–Mg alloy is discharged below 30 at% of Li, the Li^+^ diffusion coefficient in Li‒Mg anode would reduce about three orders of magnitude to ≈10^−9^ cm^2^ s^−1^.^[^ ^48^ ^]^ Therefore, the diffusion limitation will render partial loss of the specific capacity and the final discharge state is accordingly set as LiMg_2_ before the formation of *α*‐phase.^[^ ^49^ ^]^ Li–Al alloy is designed the same as the first level that the LiAl state can be fully delithiated and afford stable cycling.^[^ ^50^ ^]^ Therefore, there is no change in the Li‒Al anode.

The mass of the anode can be determined based on the assumption of the N/P ratio of 1 as follows:

(4) $$m_{\mathrm{an}}k_{\mathrm{an}}q_{\mathrm{an}}=m_{\mathrm{ca}}k_{\mathrm{ca}}q_{S}$$

where *m*_an_ and *m*_ca_ are the total mass of the anode and the S cathode, respectively, *k*_an_ and *k*_ca_ are the content of active materials in anode and S cathode, respectively, *q*_an_ is the specific capacity of the anode material, and *q*_S_ is the specific capacity of the S cathode. The content of conductive additives and binders is determined according to the values in Table 2.

As is discussed above, the criterion at the second level is to judge whether the required electrolyte amount determined by a targeted energy density is enough to infiltrate the porous electrodes in theory. Herein, we define the necessary electrolyte volume to infiltrate the electrode pores as the required electrolyte volume ($V_{\mathrm{ele}}^{\mathrm{re}}$) and the necessary electrolyte volume to reach the targeted energy density as the containable electrolyte volume ($V_{\mathrm{ele}}^{\mathrm{con}}$). For a specific anode material, if the estimated $V_{\mathrm{ele}}^{\mathrm{con}}$is higher than $V_{\mathrm{ele}}^{\mathrm{re}}$, it means that there is enough electrolyte to infiltrate the porous electrodes and meet the targeted energy density so that the anode is qualified at the second level. For the minimal infiltration of the pores, $V_{\mathrm{ele}}^{\mathrm{re}}$ shall be equal to the volume of electrode pores *V*_p_, which is determined by the electrodes’ porosity (*p*) as follows:

(5) $$V_{\mathrm{ele}}^{\mathrm{re}}=V_{p}=\frac{pV_{d}}{1-p}=\frac{p}{1-p}\sum_{i}\frac{m_{i}}{\rho_{i}}$$

where *V*_d_ is the sum of electrode components’ dense volume, *m*_i_, and *ρ*_i,_ are the mass and density of each electrode component including active materials (see Table 2), conductive additives (assuming carbon materials for all the electrodes, and *ρ*_C_ = 2.1 g cm^−3^), and binders (PVDF, *ρ*_B_ = 1.9 g cm^−3^), respectively, and the *p* values are listed in Table 2.

Moreover, the mass ratio of electrolyte to S (E/S ratio) is a more widely used indicator for Li–S batteries instead of the electrolyte volume. Therefore, we calibrated $V_{\mathrm{ele}}^{\mathrm{re}}$ to the required E/S ratio ($m_{\mathrm{ele}}^{\mathrm{re}}/m_{S}$) as follows:

(6) $$\frac{m_{\mathrm{ele}}^{\mathrm{re}}}{m_{S}}=V_{\mathrm{ele}}^{\mathrm{re}}\frac{\rho_{\mathrm{ele}}}{m_{S}}$$

where *ρ*_ele_ = 1.1 g mL^−1^ is the density of electrolyte. Using Equations (4), (5), and (6) and the design parameters in Table 2, the required electrolyte amount presented by E/S ratio ($m_{\mathrm{ele}}^{\mathrm{re}}/m_{S}$) determined for the porous S cathode is 0.66 *g*_ele_ *g*_S_^−1^ and 0.59, 0.73, and 0.85 *g*_ele_ *g*_S_^−1^ for the porous anodes of Li_4.4_Si, Li_15_Ge_4_, and Li_4.4_Sn, respectively, yet 0 for the non‐porous anodes regarding Li, Li_9_Mg, and LiAl. The required electrolyte amount for Li‒S batteries that can be therefore obtained is 1.25, 1.39, and 1.51 *g*_ele_ *g*_S_^−1^ when adopting Li_4.4_Si, Li_15_Ge_4_, and Li_4.4_Sn anodes, respectively, and 0.66 *g*_ele_ *g*_S_^−1^ when adopting Li, Li_9_Mg, and LiAl anodes.

As for determining the containable electrolyte amount to reach the targeted energy density, we also use the containable E/S ratio ($m_{\mathrm{ele}}^{\mathrm{con}}/m_{S}$) to substitute the containable electrolyte volume following the tradition of Li–S battery research. The containable E/S ratio is calculated by the targeted energy density using Equation (2), where *q*_S_ is 1450 mAh g^−1^ and the values of *U* are listed in Table 1. The total mass *m*_t_ contains three parts regarding cathode, anode, and electrolyte and is calculated using the following equation:

(7) $$m_{t}=m_{\mathrm{an}}+m_{\mathrm{ca}}+m_{\mathrm{ele}}^{\mathrm{con}}$$

Derived from Equations (2) and (7), the containable E/S ratio at a given energy density *E* is:

(8) $$\frac{m_{\mathrm{ele}}^{\mathrm{con}}}{m_{S}}=\frac{\left(\bar{U}-\bar{U_{\mathrm{de}}}\right)q_{S}}{E}-\frac{m_{\mathrm{an}}+m_{\mathrm{ca}}}{m_{S}}$$

The relationship between the containable E/S ratio $m_{\mathrm{ele}}^{\mathrm{con}}/m_{S}$ and energy density *E* is presented in Figure 2b for the use of porous anodes regarding Li_4.4_Si, Li_15_Ge_4_, and Li_4.4_Sn anodes and in Figure 2c for the use of the non‐porous anodes regarding Li, Li_9_Mg, and LiAl anodes. It can be seen that the containable E/S ratios decrease monotonically as the energy density *E* increases. For the use of Li_4.4_Si, Li_15_Ge_4_, Li_4.4_Sn, Li, Li_9_Mg, and LiAl anodes, the containable E/S ratio at 500 Wh kg^−1^ at practical electrode level is 2.83, 1.53, 0.46, 4,27, 3.83, and 1.29 *g*_ele_ *g*_S_^−1^, respectively. For the Li_4.4_Sn anode, the containable E/S ratio is lower than the required E/S ratio to indicate Li‒Sn is incapable to realize 500 Wh kg^−1^ at the second level. For the Li_15_Ge_4_ anode, the containable E/S ratio is very close to the required E/S ratio. Considering the assumption at the second level, Li‒Ge is also not a promising anode to go to the next evaluation level. In contrast, there are four anodes of Li, Li_9_Mg, LiAl, and Li_4.4_Si that have the containable E/S ratio significantly higher than the required E/S ratio to meet the demand of electrode wetting. Therefore, these four anode materials will go to the next level to evaluate the feasibility to construct practical Li‒S pouch cell with energy density of 500 Wh kg^−1^.

To truly evaluate the feasibility and potential of an anode material to realize Li–S batteries of 500 Wh kg^−1^, it is necessary to evaluate it in practical pouch cell configuration that is widely used in practice to construct high‐energy‐density devices.^[^ ^5a^ ^]^ Therefore, at the third level of evaluation, we estimate the feasibility of the above four winning anode materials at the second level to fabricate practical 500 Wh kg^−1^ Li–S pouch cells. Concretely, all the necessary components of a pouch cell are taken into consideration including separators, current collectors, and packages. Herein, we apply Celgard 2400 polypropylene (PP) membrane as the separator and the electrode materials are double‐side coated on the current collectors for maximum usage. Moreover, the N/P ratio is increased to 1.3 for a more reliable battery operation. The parameters of these components are listed in **Table** **3**, which are set as the most advantaging reported values to estimate the energy density upper limit.

**Table 3:** Parameters of Li–S pouch cells

Pouch cell parameter	Value
N/P ratio	1.3
Thickness of Cu foils, *d*_Cu_ [µm]	6
Thickness of Al foils, *d*_Al_ [µm]	8
Thickness of separator, *d*_sep_ [µm]	25
Porosity of separator, *p*_sep_ [%]	40
Areal density of separator porosity, *ρ*_sep_ [mg cm^−2^]	1.35
Mass ratio of packaging and tabs in pouch cell [wt%]	5

At this level, the mass of anode *m*_an_ follows the N/P ratio of 1.3 and can be calculated using the modified Equation (4) as follows:

(9) $$m_{\mathrm{an}}k_{\mathrm{an}}q_{\mathrm{an}}=1.3\cdot m_{\mathrm{ca}}k_{\mathrm{ca}}q_{S}$$

The evaluation at this level follows the same logic as the second level and employs similar criteria by judging whether the containable electrolyte amount for a target energy density is enough for filtration of the porous electrodes and the separators. As the porous separators are considered, the required E/S ratio is determined by using the following equation including the term of separator:

(10) $$\frac{m_{\mathrm{ele}}^{\mathrm{re}}}{m_{S}}=\frac{\rho_{\mathrm{ele}}}{m_{S}}\sum_{i}\frac{p_{i}V_{di}}{1-p_{i}}=\frac{\rho_{\mathrm{ele}}}{m_{S}}\sum_{i}\left(\frac{p_{i}}{1-p_{i}}\sum_{j}\frac{m_{j}}{\rho_{j}}\right)$$

where *V*_di_ represents the dense volume of the cathode, anode, and separator; *m*_j_ and *ρ*_j_ represent the mass and density of each component of the cathode, anode, and separator including active materials, conductive additives, and binders.

Similar to the second level, the containable E/S ratio can be calculated from Equation (2) except the total mass *m*_t_ includes additional contribution from current collectors (*m*_cc_), separators (*m*_sep_), and packages (*m*_pack_). Accordingly, Equation (8) can be rewritten as follows:

(11) $$\frac{m_{\mathrm{ele}}}{m_{S}}=\frac{\left(\bar{U}-\bar{U_{\mathrm{de}}}\right)q_{S}}{E}-\frac{m_{\mathrm{ca}}+m_{\mathrm{an}}+m_{\mathrm{cc}}+m_{\mathrm{sep}}+m_{\mathrm{pack}}}{m_{S}}$$

The terms of *m*_ca_ and *m*_an_ are in proportion with *m*_S_ according to Equation (9). Moreover, the ratios of *m*_cc_ and *m*_sep_ are dependent on the areal S loading on the current collectors. Higher areal S loading means lower proportion of the current collectors and separators within the total mass. Therefore, the calculation of the containable E/S ratio at this level needs to consider an additional variate of areal S loading (*σ*, mg cm^−2^) to address the above issues. Herein, we assume the cathode specific capacity and the average discharge voltage are not influenced by the varied areal S loading, and the ratio of *m*_S_ to *m*_cc_ or *m*_sep_ can be determined as follows:

(12) $$A=\frac{m_{S}}{\sigma }=\frac{m_{\mathrm{cc}}}{d_{\mathrm{cc}}\rho_{\mathrm{cc}}}=\frac{m_{\mathrm{sep}}}{d_{\mathrm{sep}}\rho_{\mathrm{sep}}}$$

where *A* is the electrode area, *d*_cc_ and *d*_sep_ are the thickness of current collectors and separators, respectively. Based on the assumption of *m*_pack_ taking 5% of the total mass, the containable E/S ratio can be calculated using Equations (9), (11), and (12).

**Figure 3:**
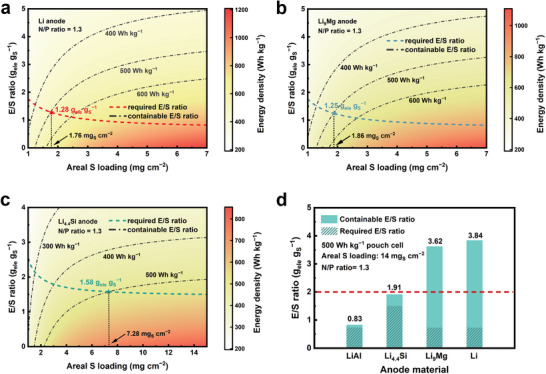
Estimated energy density in practical Li–S pouch cells at the third evaluation level. The relationship between the containable E/S ratio, the areal S loading, and the energy density using a) Li, b) Li_9_Mg, and c) Li_4.4_Si. The dot–dash lines mark the relationship between the containable E/S ratio and the areal S loading at a given energy density. The dash lines represent the required E/S ratio for cell infiltration at different areal S loadings. The intersection point of the two lines represents the minimal electrolyte amount and areal S loading to reach the target energy density. d) Comparison of the containable E/S ratio and the required E/S ratio on different anode materials to reach the energy density target of 500 Wh kg^−1^.

**Figure** **3**a,b,c demonstrates the relationship between the containable E/S ratio, the areal S loading, and the energy density by using Li, Li_9_Mg, and Li_4.4_Si anodes, respectively. As expected, the energy density increases with higher areal S loading and lower E/S ratio. The dot–dash lines in the figures represent the relationship between the containable E/S ratio and the areal S loading at a given energy density, where higher areal S loading allows more containable electrolyte. The dash lines, on the other hand, represent the relationship between the required E/S ratio and the areal S loading, where higher areal S loading allows less electrolyte volume to infiltrate the cell. The intersection points of the above two lines afford the minimal E/S ratio and areal S loading to realize the targeted energy density.

For the Li_4.4_Si anode, the minimal E/S ratio is 1.58 g_ele_ g_S_^−1^ and the minimal areal S loading is 7.28 mg cm^−2^. For the Li and Mg anodes, the minimal E/S ratio and the minimal areal S loading are much smaller to be 1.28 g_ele_ g_S_^−1^ and 1.76 mg cm^−2^ for Li, and 1.25 g_ele_ g_S_^−1^ and 1.86 mg cm^−2^ for Li_9_Mg, respectively. The above evaluation means it is more challenging for the Li_4.4_Si anode to realize Li–S pouch batteries of 500 Wh kg^−1^. Moreover, we choose a considerably high areal S loading of 14 mg cm^−2^ to determine the containable and required E/S ratio at the energy density of 500 Wh kg^−1^. Such areal S loading is a leading value in practical Li–S batteries. As shown in Figure 3d, the required E/S ratio is higher than the containable E/S ratio to indicate the infeasibility in theory as stated at the second stage. For the LiAl and Li_4.4_Si anodes, although the containable E/S ratio is higher than the required E/S ratio, the excess is very limited to suggest that their feasibility is unsatisfactory. Moreover, their containable E/S ratios are both under 2.0 g_ele_ g_S_^−1^, which is a very low electrolyte amount rarely reported in experiments. Therefore, we conclude that Li‒Al and Li‒Si are not promising anode materials to realize 500 Wh kg^−1^ Li–S pouch cells. On the contrary, the excess of the containable E/S ratio beyond the required E/S ratio on Li_9_Mg is similar to that of Li. Also, the required E/S ratios are 3.62 and 3.84 g_ele_ g_S_^−1^ for Li_9_Mg and Li, respectively, both of which have been reported in practical working Li–S pouch cells. Therefore, it is concluded that Li‒Mg is a promising alternative anode material to substitute Li metal anode to construct 500 Wh kg^−1^ Li–S pouch cells.

The above three‐level evaluation affords Li_9_Mg as a promising anode candidate besides Li metal anode to potentially realize 500 Wh kg^−1^ Li–S pouch cells, while the other eight anode materials are less attractive. Based on the above conclusion, we will afford the basic knowledge on Li–Mg alloy and review the recent research on using Li–Mg alloys in rechargeable batteries especially in the Li–S system in the next section.

## 3 Review on Alternative Anode Materials for 500 Wh kg^−1^ Li–S Batteries

**Figure 4:**
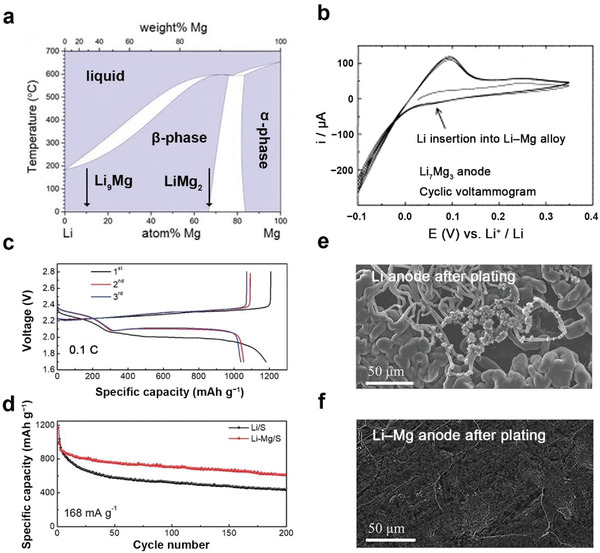
Review on Li–Mg alloy anodes in Li–S batteries. a) Calculated binary Li–Mg phase diagram. Reproduced with permission.^[^ ^48^ ^]^ Copyright 2019, Wiley‐VCH. b) Cyclic voltammograms of Li_7_Mg_3_ in nonaqueous electrolyte to demonstrate its stability. Reproduced with permission.^[^ ^28^ ^]^ Copyright 2001, Elsevier. c) Charge–discharge profiles of Li–S batteries with Li–Mg alloy anodes. d) Cycling performances of Li and Li–Mg anode in Li–S batteries. SEM images of e) Li metal anode and f) Li–Mg alloy anode after Li plating at 0.5 mA cm^−2^ for 24 h. Reproduced with permission.^[^ ^56^ ^]^ Copyright 2019, Wiley‐VCH.

The first attempt of using Li–Mg alloy as electrode in nonaqueous electrolyte dates back to the early 1970s, when Dey demonstrated that Li can be electrochemically alloyed with other metals in organic electrolytes and Mg is one of the candidates with the lowest alloying potential versus Li/Li^+^.^[^ ^23a^ ^]^ Using thermochemical analysis and electrical conductivity measurement, Grube et al. presented the binary phase diagram of Li‒Mg alloy in the early 1930s (**Figure** **4**a).^[^ ^27^ ^]^ The microstructure of Li‒Mg alloy depends on the Li content. From 0 to 15 at% of Li, the Li‒Mg alloy exists in *α*‐phase with a hexagonal close‐packed (hcp) structure. From 15 at% to 32 at% of Li, there is a mixed *α* + *β* phase, where the *β* phase is a body cubic (bcc) structure. From 32 at% to 100 at% corresponding to nearly LiMg_2_ to pure Li, the Li‒Mg alloy exists in the Li‐rich *β*‐phase, which is the same structure as Li metal.^[^ ^51^ ^]^ The similar structure as Li metal makes the Li‒Mg alloy to avoid phase change and maintain the stable scaffold under electrochemical working conditions to permit reversible Li^+^ insertion and removal.^[^ ^48^, ^52^ ^]^ The *β*‐phase alloy also demonstrates good room‐temperature ductility and cold formability to allow the straightforward fabrication of foil electrodes by simple rolling.^[^ ^49^, ^53^ ^]^ Nevertheless, massive production of the *β*‐phase Li–Mg alloy anode still requires further efforts to promote its application in batteries.^[^ ^54^ ^]^

Following the structure identification, the electrochemical performance of the Li‒Mg alloy anode was investigated by Kim et al. It was found that the delithiation and lithiation potential of Mg was very low to be 15 mV during charging and 50 mV during discharging versus Li/Li^+^, respectively.^[^ ^55^ ^]^ Moreover, Zhong et al. found that the tendency of Li dendrite growth on Li–Mg alloy surface can be sufficiently suppressed even if the potential decreased to −0.1 V versus Li/Li^+^ (Figure 4b).^[^ ^28^ ^]^ The cyclic voltammograms also revealed the high diffusivity of Li^+^ being ≈10^−7^ cm^2^ s^−1^ in *β*‐phase Li–Mg alloy electrodes, which is two or three orders of magnitude higher than other Li‐alloy systems, suggesting that the Li–Mg alloy is potentially advantageous to realize high‐rate cycling without dendrite formation.^[^ ^28^ ^]^ However, it shall be noted that once the phase transition from the *β*‐phase to the *α*‐phase happens under practical working conditions, the low Li diffusion coefficient in the *α*‐phase would result in a diffusion‐limited delithiation termination and consequently reduced capacity in the following cycles.^[^ ^47^ ^]^ This issue can be addressed by optimizing the alloy anode structure and controlling the operation conditions, where more efforts are needed in this direction.^[^ ^49^ ^]^

The Li–Mg alloy anode has been evaluated in Li–S batteries to demonstrate its potential to realize stable anode cycling. Kong employed Li_9.5_Mg_0.5_ as the anode to substitute Li metal anode and evaluated the battery performances.^[^ ^56^ ^]^ An obvious overpotential was observed on the discharge curve of the first cycle of the Li–Mg | S battery at 0.1 C, which was assigned to partial passivation by the formation of an inactive MgO layer on the pristine Li–Mg alloy surface (Figure 4c). The discharge overpotential was generally overcome at the following cycles after initial activation, and the dense MgO layer converted to a porous and robust passivation layer to inhibit further corrosion between the anode and electrolyte. In the following 200 cycles, the Li–Mg | S battery afforded higher capacity retention than the control cell to suggest higher anode stability (Figure 4d). Morphology evaluation using scanning electron microscope (SEM) demonstrated a smooth surface of the Li–Mg alloy anode while massive Li dendrites were observed on the Li metal anode (Figure 4e,f). However, it shall be noted that the above evaluation is conducted under mild working conditions using low areal S loading of 2 mg cm^−2^ and thick anode (basically N/P ratio = 7). Therefore, although the Li–Mg alloy anode demonstrated improved stability, the performance shall be further evaluated in practical high‐energy‐density Li–S pouch cells under working conditions to reveal the actual performance promotion capability. To this end, systematic experimental research on using the Li–Mg alloy as the anode to construct 500 Wh kg^−1^ Li–S batteries is highly encouraged.

**Figure 5:**
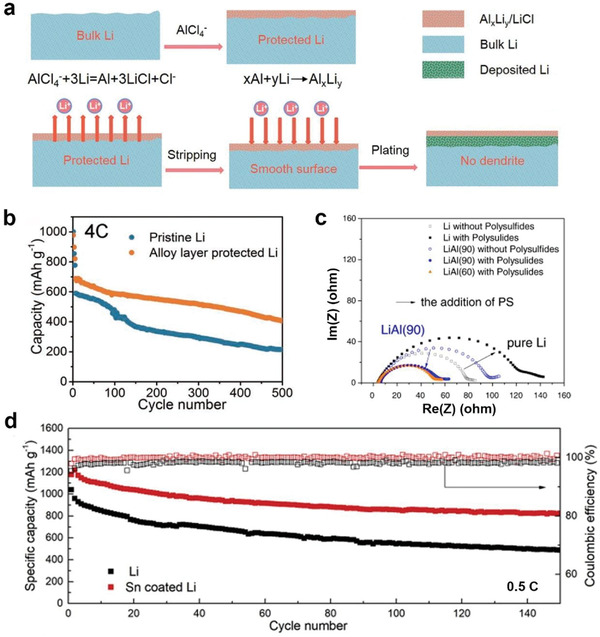
Review on protective alloy coating on Li metal anode. a) Schematic illustration of constructing an Al–Li alloy protective layer on Li metal anode by using ionic liquids. b) Cycling performance of Li–S batteries at 4 C with the Al–Li alloy protected anode. Reproduced with permission.^[^ ^57^ ^]^ Copyright 2020, Wiley‐VCH. c) EIS spectra of Li‐based anodes with Al protective layer at different lamination temperatures. Reproduced with permission.^[^ ^58^ ^]^ Copyright 2013, Elsevier. d) Cycling performance of Li‒S batteries at 0.5 C with Sn coated Li anode. Reproduced with permission.^[^ ^59^ ^]^ Copyright 2020, Elsevier.

Despite the other eight anode materials not passing the three‐level evaluation, their advantages in structural stability under working conditions shall be given serious attention. Compositing these anode materials with Li metal can potentially improve the cycling stability of the composite anode even if they are in separate phases. There have been experimental researches on employing the structurally stable Li‐based alloys as a protective layer on Li metal anode surface to inhibit Li dendrite growth in Li–S batteries. For instance, Lu et al. fabricated Li–Al alloy‐based protective layer on Li metal anode (**Figure** **5**a).^[^ ^57^ ^]^ The Li metal was treated with ionic liquid AlCl_3_/[EMIm]Cl to form a 10 µm thick protective Li‒Al alloy layer through in situ reaction. The mechanical modulus of this Li‒Al layer was ≈1.4 times higher than the pristine SEI, and the ion conductivity was more than 100 times of the pristine SEI. The Li–S batteries with the Li‒Al alloy protected anode performed higher capacity retention and more stable Coulombic efficiency than the control batteries. High‐rate performances up to 4 C of the Li‒Al alloy protected anode were also realized (Figure 5b). Similarly, Kim et al. proposed a lamination method of coating a thin Al layer on Li metal anode surface at elevated temperature.^[^ ^58^ ^]^ The Li‒Al layer was 0.8 µm in experiment, and the anodes with the Li–Al layer showed reduced charge transfer resistance compared with bulk Li under the existence of polysulfides (Figure 5c). Additional studies revealed that the alloy anode was more effective in protecting Li metal anode in electrolyte with LiNO_3_ than the original SEI formed with ether‐based electrolyte.

Sn coating on Li foil is also effective in working Li‒S batteries. Xia et al. fabricated a 250 nm Sn protective layer on Li metal anode by thermal evaporation.^[^ ^59^ ^]^ The fabricated layer demonstrated a decreased overpotential of Li^+^ diffusion and improved cycle performance in symmetric cells.^[^ ^59^ ^]^ Theoretical simulation also revealed the migration energy barrier was as low as 0.3 eV, proving the facilitated Li^+^ transport at the interface between the Li–Sn layer and electrolyte. The Li–Sn alloy coated Li anode was further verified in Li‒S batteries and demonstrated reduced capacity decay compared to bare Li anode, and the Coulombic efficiency maintained almost 100% during 150 cycles (Figure 5d). Inspired by the above works, constructing a protective layer using the structurally stable Li‐based alloys on the surface of Li metal anode paves an additional pathway to improve the cycling stability of the anode while maintaining the potential to reach actual high energy density in Li–S batteries. Nevertheless, the content of the protect layer shall be seriously controlled and the protection mechanism, as well as the rational design of the composite anode structure, requires further investigation and evaluation under practical working conditions.

## 4 Conclusion and Perspective

The intrinsic advantage of Li–S batteries lies in the ultrahigh theoretical energy density and the potential to realize high‐energy‐density devices to meet the growing demand for energy storage. The Li metal anode makes the core contribution to high energy density due to the ultrahigh specific capacity of 3860 mAh g^−1^ and ultralow potential of −3.040 V versus the standard hydrogen electrode. However, the high chemical reactivity and poor structural stability of Li metal anode endow disadvantages of very limited cycling lifespan in high‐energy‐density Li–S pouch cells. Based on the above consideration, it is necessary to evaluate the potential and feasibility of alternative anode materials to realize high‐energy‐density Li–S batteries. This contribution proposes a systematic three‐level quantitative evaluation method to estimate the potential of the anode candidates to realize a targeted energy density. At the first level, the theoretical energy density is calculated to estimate the upper limit of the anode materials. Following that, necessary electrode components and electrolyte for electrode filtration are taken into consideration at the second practical electrode level. Finally, the anode materials are evaluated in working pouch cell configuration to estimate the feasibility in practice. Aiming at the targeted energy density of 500 Wh kg^−1^, the Li–Mg alloy anode outperforms other Li‐based alloys to demonstrate promising potential besides the Li metal anode. The Li_9_Mg anode is predicted to potentially afford high energy density and long cycling stability in working Li–S batteries.

Based on the above evaluation results, we propose three perspectives on developing substitute anode materials beyond Li metal anode in high‐energy‐density Li–S batteries. The first issue is that the mechanism of alloy anodes regarding lithiation and delithiation shall be systematically investigated. The basic behaviors of the structure evolution and the underlying chemistry of why the alloy anodes are more stable than Li metal anode need to be revealed. To this end, in situ and operando characterizations as well as multiscale simulation are expected to afford in‐depth understanding combining with experiments. The mechanism investigation will lead the direction for rational design of advanced anode materials. The second issue is to understand the structure and properties of the SEI on alloy anodes since the SEI plays a determining role in electrochemical performances. The interactions between alloy anodes and solvents, salts, and additives need to be clarified. And, the SEI formation kinetics require detailed investigation. Furthermore, developing advanced electrolytes that are compatible with alloy anodes will further promote the cycling stability of Li–S batteries. Notably, electrolyte design in Li–S batteries requires full consideration from the cathode side that massive polysulfides are supposed to be dissolved to support the cathode conversion reactions. Lastly, it shall be emphasized that the performances of alloy anodes shall be evaluated in high‐energy‐density Li–S pouch cells under practical working conditions to reveal the actual feasibility. Evaluation under such rigorous conditions will reveal the actual performances and true challenges needed to be addressed. To this end, the design parameters of the cells shall be afforded for more reliable comparison among the anodes and strategies.

In summary, Li–Mg alloy anode is selected as a promising anode material to potentially realize 500 Wh kg^−1^ Li–S batteries besides Li metal anode based on the three‐level evaluation proposed in this manuscript. Although the exclusive use of other Li‐based anode materials is not promising to realize high energy density, their composition with Li metal to fabricate composite anode is feasible to address the challenges of Li metal anode. There have been several pioneer researches on using alloy anode materials to substitute Li metal anode in Li–S batteries. It is believed the development of advanced Li‐based anode will make significant contribution to realizing long‐cycling 500 Wh kg^−1^ Li–S batteries and beyond.

## Conflict of Interest

The authors declare no conflict of interest.
